# Stumps of *Eucalyptus globulus* as a Source of Antioxidant and Antimicrobial Polyphenols

**DOI:** 10.3390/molecules191016428

**Published:** 2014-10-13

**Authors:** Ângelo Luís, Duarte Neiva, Helena Pereira, Jorge Gominho, Fernanda Domingues, Ana Paula Duarte

**Affiliations:** 1 Centro de Investigação em Ciências da Saúde, Universidade da Beira Interior, Av. Infante D. Henrique, Covilhã 6200-506, Portugal; 2 Centro de Estudos Florestais, Instituto Superior de Agronomia, Universidade de Lisboa, Tapada da Ajuda, Lisboa 1349-017, Portugal

**Keywords:** *Eucalyptus globulus*, stumps, polyphenols, antioxidant activity, antimicrobial properties

## Abstract

These past years have seen an enormous development of the area of natural antioxidants and antimicrobials. *Eucalyptus globulus* is widely cultivated in subtropical and Mediterranean regions in intensive short rotation coppice plantations. In the Portuguese context, *E. globulus* is the third species in terms of forest area. The stump is the basal part of the tree, including the near-the-ground stem portion and the woody roots that remain after stem felling. The purpose of this work was to study the phytochemical profile and to evaluate the antioxidant and antimicrobial properties of several crude stump wood and stump bark extracts of *E. globulus*, comparing it with similar extracts of *E. globulus* wood (industrial chips). The results showed the presence of high concentrations of total phenolic compounds (>200 mg GAE/g extract) and flavonoids (>10 mg QE/g extract) in *E. globulus* stump extracts. Generally the stump wood extracts stands out from the other ones, presenting the highest percentages of inhibition of linoleic acid oxidation. It was also possible to conclude that the extracts were more active against Gram-positive bacteria, presenting low MIC values. This study thus provides information supporting the economic valorization of *E. globulus* stump wood.

## 1. Introduction

These past years have seen an enormous development of the area of natural antioxidants due to the increasing limitations on the use of synthetic antioxidants and enhanced public awareness of health issues [1]. Thus, research has focused on the identification of novel antioxidants from natural sources [2,3]. Plant phenolic compounds such as flavonoids, sterols, lignin phenols, and various terpene-related compounds are all potent antioxidants [4]. Many of these compounds have therapeutic properties and are known for their anticarcinogenic, antimutagenic, cardioprotective, antineurodegenerative and antimicrobial activities [4,5,6,7]. Numerous studies have been carried out to obtain antioxidants from several plant materials such as fruits, vegetables, herbs and spices, agricultural by-products, and different tree materials [2,8,9,10]. Antioxidant activities (measured as the ability to scavenge free radicals) have been reported for cell-wall polysaccharides from a variety of wood sources, including xylan, 4-*O*-methyl-d-glucurono-d-xylan, galactomannan and galactoglucomannan [2,8,9,10].

On the other hand, the increasing resistance of pathogens towards antibiotics presents a major threat to public health because it reduces the effectiveness of antibiotic treatment, which could lead to an increase in morbidity and mortality [11,12,13]. Concerning nosocomial infections, methicillin-resistant *Staphylococcus aureus* (MRSA) and vancomycin-resistant enterococci (VRE) are the most important resistant pathogens among Gram-positive bacteria [14,15]. The emergence of resistance in Gram-negative bacteria (*Klebsiella pneumoniae*, *Escherichia coli*, *Pseudomonas aeruginosa*, and *Acinetobacter baumannii*) has already been documented [16,17]. Many antibiotics that were frequently used in the past have become less effective against these pathogens. Hence, there is an urgent need to find alternative antimicrobial agents to treat resistant pathogenic microorganisms [16]. Although the most important sources of antibiotics are molds, actinomycetes and bacteria, higher plants also contain many classes of secondary metabolites with this property [18], thus, efforts have been made to evaluate the antimicrobial activity of a wide array of natural products, including plant metabolites, in order to isolate and characterize novel compounds which could inhibit bacteria and fungi, or even serve as models for new molecules [18].

*Eucalyptus globulus* is widely cultivated in subtropical and Mediterranean regions in intensive short rotation coppice plantations as the main fiber source for the pulp and paper industry [3,19]. In the Portuguese context, *E. globulus* is the third species in terms of forest area (about 672,000 ha), representing nearly 31% of the *E. globulus* area planted worldwide [20]. These activities generate large amounts of biomass residues (leaves, bark and stumps) which are either left in the field or burned for energy production [20,21].

The stump is the basal part of the tree, including the near-the-ground stem portion and the woody roots that remain after stem felling [21]. In coppice systems, the stump is the oldest part of the tree, therefore enriched in heartwood, reaction wood and cicatricial tissues, and with a higher content in extractives and polysaccharides. In the past, the stumps were typically uprooted and destroyed, and the resulting biomass spread or incorporated into the soil [21]. Nowadays, stumps have become a significant biomass source for heat and power generation, due to the scarcity of other biomass types and the decreasing cost of grinding and screening operations, and their removal from forest plantations to use as a bioenergy source or as raw material for the extraction of bioactive molecules has led to a strong discussion concerning the effects of these practices on soil quality and forestry sustainability [21]. Thus, from the biorefinery perspective, the stumps can be used as raw material for the extraction of bioactive molecules with antioxidant and antimicrobial properties, namely polyphenolic compounds.

In this context, the purpose of this work was to study the phytochemical profile (total phenolic compounds, tannins and flavonoids) and to evaluate the antioxidant and antimicrobial properties (studied by disc diffusion and resazurin microtiter assays) of several crude extracts of *E. globulus* stump wood and stump bark, comparing it with similar extracts of *E. globulus* wood (industrial chips). It is noteworthy to emphasize that, as far as we know, the phytochemical profile of *E. globulus* stumps is reported here for the first time.

## 2. Results and Discussion

In a continuous search for new plant-derived bioactive compounds and considering the biomass from stumps of *Eucalyptus globulus*, in this work it was decided to investigate the potential of this raw material as a source of molecules, namely phytochemicals, with antioxidant and antimicrobial activity. In this sense, the influence of four solvents (*n*-hexane, ethanol, methanol and 75% aqueous ethanol) in the extraction of bioactive phenolic compounds from wood, stump wood and stump bark of *E. globulus* was studied. Then, the antioxidant and antimicrobial activities of these extracts were screened. Concerning the extraction yields (Table 1), generally, the stump wood extracts have higher values (>8%), when compared with the wood and stump bark extracts, which indicates that stumps are a potential source of bioactive molecules. Methanolic extracts presented higher extraction yields, particularly in the case of stump wood extracts (12.30%).

**Table 1:** Extraction yields of the *E. globulus* extracts.

Solvent	*Eucalyptus globulus* Parts	Extraction Yields (%)
*n*-Hexane	Wood	1.00
	Stump wood	0.42
	Stump bark	2.70
Ethanol	Wood	1.00
	Stump wood	9.31
	Stump bark	7.41
Methanol	Wood	1.70
	Stump wood	12.30
	Stump bark	9.92
75% Ethanol	Wood	3.00
	Stump wood	8.07
	Stump bark	5.88

### 2.1. Phytochemical Profile

The production of free radicals, including reactive oxygen species (ROS), is an inevitable phenomenon associated with an aerobic lifestyle. A prerequisite for the function and development of cells in an oxygen-containing environment is the presence of protective systems involving specialized enzymes and low-molecular-weight antioxidants [22]. Phenolic compounds seem to be efficient nonenzymatic protectors against oxidative stress. They can act as antioxidants in a variety ways, preventing transition metal ions from initiating oxidation, quenching the oxidation intermediates (including ROS), and inhibiting various prooxidant enzymes [22]. Phenols are also able to donate the hydrogen atoms of the phenolic OH groups to free radicals, thus stopping the propagation chain during the oxidation process [22]. Thus, the determination of various classes of phenolic compounds is fundamental to better understand the biological activities of extracts of vegetal origin. In the present study, the phenolic compounds present in the extracts were determined with Folin-Ciocalteu’s reagent (Table 2). This assay involves the oxidation in alkaline solution of phenols by the yellow molybdotungstophosphoric acid heteropolyanion reagent and colorimetric measurement of the resultant molybdotungstophosphate blue [22]. Except for *n*-hexane extracts, stump wood extracts presents the highest concentration in phenolic compounds, when compared with wood and stump bark extracts for each solvent. Moreover, the phenolic content in all the extracts was superior than in other extracts studied by our research group [23,24], which indicates the potential of the extracts now studied, particularly the stump ones, as a potential source of phenolic molecules for medicinal purposes.

**Table 2:** Phytochemical composition of the *E. globulus* extracts (* Mean ± standard deviation; ^#^ Modal values).

Solvent	*Eucalyptus globulus* Parts	Total Phenolics (mg GAE/g Extract) *	Taninns (mg GAE/g Extract) ^#^	Flavonoids (mg QE/g Extract) *
*n*-Hexane	Wood	17.00 ± 2.55	17.00	59.23 ± 0.58
	Stump wood	25.93 ± 0.31	13.40	47.16 ± 2.27
	Stump bark	45.80 ± 2.88	13.93	58.34 ± 2.18
Ethanol	Wood	262.67 ± 3.06	N/D	11.90 ± 0.60
	Stump wood	460.00 ± 5.61	N/D	33.61 ± 2.58
	Stump bark	253.07 ± 4.94	N/D	8.83 ± 1.32
Methanol	Wood	251.00 ± 5.06	N/D	12.89 ± 0.59
	Stump wood	451.10 ± 4.10	N/D	43.06 ± 3.16
	Stump bark	233.90 ± 2.12	N/D	8.50 ± 0.49
75% Ethanol	Wood	218.67 ± 4.52	N/D	11.78 ± 0.80
	Stump wood	444.60 ± 2.55	N/D	44.87 ± 1.56
	Stump bark	382.50 ± 4.10	39.97	12.13 ± 0.11

N/D-Not Detected.

It was observed that tannins are significantly present in *n*-hexane extracts (Table 2). This fact can be explained by the low polarity of tannins, due to their large chemical structure, which favor their extraction by nonpolar solvents like *n*-hexane. In addition, for the *n*-hexane extract of wood, the total amount of phenolic compounds corresponds to tannins. Hydroalcoholic extract of stump bark also presents a significant amount of tannins. These compounds are high molecular weight (>3000 Da) phenolic compounds that bind to proteins to form complexes which precipitate dietary feed nutrients such as carbohydrates, proteins and minerals [25]. Tannins have been reported to exert other physiological effects, e.g., they can reduce blood pressure, accelerate blood clotting, decrease serum lipid levels, modulate immunoresponses and also produce liver necrosis [26], which justifies their determination.

Flavonoids are diphenylpropanes that commonly occur in plants and are frequent components of the human diet [27]. The immediate flavonoid family members include flavones, isoflavones, and the 2,3-dihydroderivatives of flavone, namely flavanones, which are interconvertible with the isomeric chalcones [27]. Flavanones undergo a series of transformations affecting the heterocyclic C ring to give rise to other family members of flavonoids, including anthocyanins and catechins [27]. Some flavonoids have been found to possess several biological activities, and for that reason, their quantification in vegetal extracts is important. The flavonoids present in the extracts were determined with the aluminum chloride colorimetric method (Table 2). Once again, the stump wood extracts stand out among the other raw material extracts, because of their higher concentrations of flavonoids. Moreover the results now obtained for flavonoids content were much superior than for other vegetal extracts [28,29]. The difference between the results of flavonoids extracted with *n*-hexane and by the other polar solvents (ethanol, methanol and 75% ethanol), may be explained by the fact that flavonoids can be linked to sugar molecules, in the form of heterosides. Heterosides were more easily extracted by polar solvents and free-flavonoids (aglycones) can be simply extracted by non-polar solvents like *n*-hexane. Therefore, the *n*-hexane extracts are rich in aglyconic flavonoids contrariwise to the other polar extracts which present more heterosidic flavonoid molecules.

### 2.2. Antioxidant Activity

In recent years there has been an increasing interest in natural antioxidant compounds. Antioxidants seem to play an important role as a health-protecting factor. Scientific evidence suggests that antioxidants reduce the risk of several chronic diseases including coronary diseases and cancer. Although the protective effects have been primarily attributed to the well-known antioxidants, such as vitamin C, vitamin E, and β-carotene, plant phenolics may also play a significant role [30]. Hence, the study of antioxidant activity of plant extracts in order to identify new natural sources of antioxidant molecules is useful. The antioxidant activity of *E. globulus* extracts was evaluated by the 2,2-diphenyl-1-picrylhydrazyl (DPPH) method (Table 3). DPPH, a paramagnetic compound with an odd electron, shows strong absorption band at 517 nm in methanol. The absorbance decreases as a result of color change from purple to yellow due to the scavenging of free radical by antioxidants through donation of hydrogen to form the stable DPPH–H molecule [31].

Overall, with the exception of the *n*-hexane ones, all the extracts presented very strong antioxidant activity, as their Antioxidant Activity Index (AAI) values were higher than 2.0 [32]. Antioxidant molecules obtained from natural sources (plant extracts or essential oils), typically molecules with phenolic structures, are more often extracted by polar solvents, like alcoholic or aqueous mixtures of solvents. This fact can explain why the polar extracts of *E. globulus* presented higher antioxidant activity when compared to the ones obtained using *n*-hexane, a non-polar solvent.

Particularly the antioxidant activity of the extracts from the stump wood exceeds the values obtained for the wood and the stump bark ones, in terms of antioxidant activity. This finding is very relevant because it is the first time that the antioxidant activity of *E. globulus* stumps has been studied. Moreover, these results provide suggestions for the economic valorization of such biomass, constituting the first step into the exploitation of antioxidant properties of extracts obtained from *E. globulus* stumps. As the stumps are the oldest part of the tree, they are rich in bioactive compounds, like phenolics, with antioxidant properties. The results obtained for the parameters of antioxidant activity for the standard compounds are very similar to those determined by the same method by other researchers [9,32].

**Table 3:** Antioxidant properties of *E. globulus* extracts measured by DPPH method (* Mean ± standard deviation).

Solvent	*Eucalyptus globulus* Parts	IC_50_ (mg/L) *	Antioxidant Activity Index (AAI) *	Antioxidant Activity
*n*-Hexane	Wood	369.29 ± 23.62	0.17 ± 0.06	Poor
	Stump wood	189.91 ± 6.38	0.25 ± 0.07	Poor
	Stump bark	170.29 ± 7.43	0.26 ± 0.03	Poor
Ethanol	Wood	9.33 ± 0.61	4.94 ± 0.13	Very Strong
	Stump wood	5.97 ± 0.27	7.39 ± 0.37	Very Strong
	Stump bark	11.32 ± 0.63	3.85 ± 0.27	Very Strong
Methanol	Wood	10.84 ± 0.23	4.62 ± 0.27	Very Strong
	Stump wood	6.00 ± 0.26	7.46 ± 0.16	Very Strong
	Stump bark	12.47 ± 0.66	3.58 ± 0.16	Very Strong
75% Ethanol	Wood	17.29 ± 1.14	2.60 ± 0.12	Very Strong
	Stump wood	6.35 ± 0.19	8.80 ± 0.27	Very Strong
	Stump bark	8.61 ± 0.29	6.30 ± 0.46	Very Strong
Gallic Acid		2.23 ± 0.02	22.77 ± 0.25	Very Strong
Quercetin		4.32 ± 0.39	12.17 ± 1.71	Very Strong

Another interesting result was obtained for the hydroalcoholic extract of stump bark, that has a very small IC_50_ value which in turn results in a high AAI value and thus very strong antioxidant activity. Relative to the solvent used, it seems that there is no difference between the use of ethanol, methanol or 75% ethanol as all of these solvents produced similar AAI results. When compared with previous results for other raw materials [23], the ones obtained in the present work are much more promising in what concerns antioxidant properties, indicating that stumps are a good potential source of molecules with biological activity.

A positive linear correlation between AAI and total phenolic content was observed for all the extracts studied (R^2^ = 0.9636). These results indicate that phenolic compounds could be the main contributors to the antioxidant properties of these extracts. This result is in agreement with several of our previous studies [23,24,28,29]. This conclusion is explained by the molecular structure of phenolic compounds which enables them with redox properties, and so, to act as reducing agents, hydrogen donors, and singlet oxygen quenchers [33]. The same analysis was conducted for flavonoids and it was verified that there was no correlation (R^2^ = 0.0758) between these compounds and the antioxidant activity of the extracts. It is known that only flavonoids of a certain structure and particular the hydroxyl position in the molecule determine its antioxidant properties; in general these properties depend on the ability to donate either hydrogen or an electron to a free radical [33].

**Figure 1:**
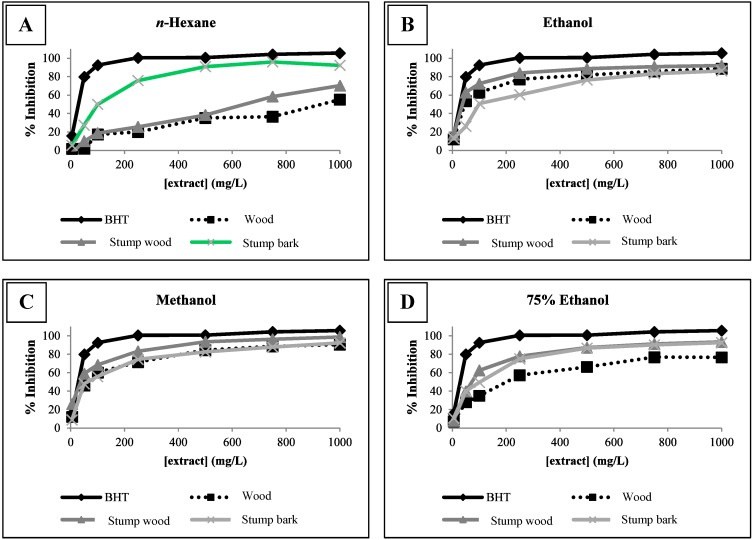
Antioxidant activity of *E. globulus* extracts measured by β-carotene bleaching test. (**A**) *n*-Hexane extracts. (**B**) Ethanolic extracts. (**C**) Methanolic extracts. (**D**) Hydroalcoholic extracts.

One problem in the determination of the antioxidant activity is that this activity is variable and depends on the method used. It is known that the antioxidant mechanism in different biological matrices is very complex and many other factors may be involved in this mechanism [34]. Given this complexity, the use of only one method to determine the antioxidant activity of the extracts is not sufficient to reach a conclusion. For this reason, we have used two different methods and diverse antioxidant properties can then be determined. Besides the DPPH method, the antioxidant activity of the extracts was also evaluated by the β-carotene bleaching test (Figure 1). This test was selected for antioxidant activity determination because it gives the capacity of lipid peroxidation inhibition of the extracts and is also useful in terms of food industry, as it is carried out in an emulsion, a situation frequent in foods. On the other hand, it is generally agreed that the oxidation is initiated by free radical attack; therefore, assays to evaluate the radical scavenging activity are representative of the potential of a compound to retard oxidation [35].

The results of the antioxidant properties for the *E. globulus* extracts, in terms of percentage of inhibition of linoleic acid molecules oxidation, are presented in the Figure 1. Generally the stump wood extracts stands out from the other ones, presenting the highest percentages of inhibition, closely similar to those of the synthetic antioxidant BHT, used as positive control.

In the case of *n*-hexane extracts, it was the stump bark extract that had presented the biggest capacity to inhibit the oxidation of linoleic acid. It seems that ethanol and methanol are more appropriate solvents to extract molecules which inhibit lipid peroxidation.

With the results now obtained, it is possible to conclude that these extracts, particularly the stump ones, are a source of bioactive compounds with the capacity to inhibit lipid peroxidation, which indicates the potential of these extracts to be used as food preservatives by the food industry, in the preservation of foods with high fat content. Future work is needed in order to verify the toxicity and bioavailability of the compounds present in these extracts in what concerns its possible applications on foods.

### 2.3. Antimicrobial Activity

In the present scenario of emerging multiple drug resistance to human pathogenic organisms, the search for new antimicrobial substances from other sources, including plants, as received great attention [36]. In this work, the antimicrobial activity of *E. globulus* extracts against several strains of human infecting microorganisms was evaluated. The diameters of inhibition zones for the studied extracts, measured by the disc diffusion assay, are presented in Table 4. It was observed that Gram-positive bacteria, including clinical isolates of *S. aureus* and MRSA strains, were more susceptible to the action of the extracts, presenting the biggest inhibition zones, contrary to which was observed for Gram-negative strains, namely *E. coli* and *P. aeruginosa*. Moreover, yeasts strains were also susceptible to the extracts, particularly the strain of *C. tropicalis*. It is emphasized that in this study antimicrobial activity against yeasts and Gram-negative bacteria, which are often more resistant to the action of extracts from natural origin, was found [37,38].

The results obtained by the disc diffusion method indicate the potential use of these extracts as antimicrobials. Following the screening of antimicrobial properties of *E. globulus* extracts, the Minimum Inhibitory Concentration (MIC) values of the extracts were determined by the resazurin microtiter assay and the results are presented in Table 5. Despite the fact of some extracts did not present inhibition zones in the disc diffusion assay, they presented low MIC values, and consequently good antimicrobial properties, this may be due to the weak diffusion of the extracts in the agar plates, which influences the results obtained using the disc diffusion assay. The *n*-hexane extracts were not suitable for MIC determination because of their poor solubility in the (water-based) culture medium. The majority of the studied extracts presented low MIC values against several strains of microorganisms, particularly the strains of *S. aureus*, both reference and clinical isolates (including MRSA strains), food-borne pathogens (*B. cereus* and *L. monocytogenes*), *Candida* strains and for some Gram-negative bacteria. The action of *E. globulus* extracts is also dependent of the strain, because there are significant differences within the same genus of microorganism, which can be observed in the particular case of *S. aureus* strains, where is possible to detect that SA 03/10 and MRSA 12/08 presented very high MIC values, suggesting its low susceptibility to the extracts.

Accumulation of certain plant secondary metabolites is induced upon pathogen attack and these metabolites are referred to as phytoalexins. Some phytoalexins have broad spectrum activities against a wide range of pathogens, while others target specific pathogens [1,2,3,4,5,6,7,8,9,10]. Phenolics are a class of plant secondary metabolites that contain one or more hydroxyl derivatives of benzene rings and are widely distributed in plants and are used for defensive functions in many plant species [1,2,3,4,5,6,7,8,9,10]. Phenolics in plants are mainly synthesized from the phenylpropanoid pathway. *In vitro* antimicrobial activities of the phenylpropanoid pathway intermediates, including *p*-coumaric acid, caffeic acid, ferulic acid and sinapic acid, and pathway derivatives, including flavonoid aglycones and glycosides, have been demonstrated [1,2,3,4,5,6,7,8,9,10]. Phenolic compounds with less complex structures, such as catechol and coumarin, have also shown to exhibit bactericidal and fungicidal activities. Increased accumulation of phenolic phytoalexins in plants can promote host defense against pathogens [1,2,3,4,5,6,7,8,9,10].

**Table 4:** Diameter of inhibition zones (mm) of *E. globulus* extracts (Mean ± standard deviation).

Strains	*n*-Hexane			Ethanol			Methanol			75% Ethanol			Controls	
	Wood	Stump Wood	Stump Bark	Wood	Stump Wood	Stump Bark	Wood	Stump Wood	Stump Bark	Wood	Stump Wood	Stump Bark	DMSO	Tetracycline
**Gram-positive Bacteria**														
*S. aureus* ATCC 25923	13.00 ± 0.00	10.00 ± 0.00	16.50 ± 0.71	20.50 ± 0.71	21.00 ± 0.00	20.50 ± 0.71	16.50 ± 0.71	21.00 ± 1.41	20.00 ± 0.00	20.50 ± 0.71	22.50 ± 0.71	22.50 ± 0.71	6.00 ± 0.00	30.25 ± 0.50
*B. cereus* ATCC 11778	8.00 ± 0.00	10.50 ± 0.71	20.50 ± 0.71	15.00 ± 1.41	16.00 ± 1.41	19.00 ± 1.41	15.50 ± 0.71	17.00 ± 1.41	18.00 ± 0.00	17.50 ± 0.71	16.00 ± 1.41	19.00 ± 0.00	6.00 ± 0.00	30.00 ± 0.82
*L. monocytogenes* LMG 16779	6.50 ± 0.71	7.00 ± 1.41	17.00 ± 1.41	22.50 ± 2.12	23.00 ± 1.41	21.00 ± 1.41	17.50 ± 0.71	27.50 ± 2.12	20.00 ± 1.41	24.50 ± 0.71	30.00 ± 0.00	20.00 ± 0.00	6.00 ± 0.00	18.25 ± 0.60
*E. faecalis* ATCC 29212	6.00 ± 0.00	6.00 ± 0.00	17.00 ± 1.41	11.00 ± 0.00	10.00 ± 0.00	14.00 ± 0.00	9.50 ± 0.71	11.00 ± 1.41	15.00 ± 0.00	10.00 ± 0.00	11.50 ± 0.71	15.50 ± 0.71	6.00 ± 0.00	25.20 ± 0.58
*S. aureus* SA 01/10	6.00 ± 0.00	6.00 ± 0.00	11.50 ± 0.71	13.50 ± 0.71	15.50 ± 0.71	14.00 ± 0.00	14.00 ± 1.41	14.50 ± 0.71	16.50 ± 0.71	13.50 ± 0.71	16.00 ± 1.41	18.50 ± 0.71	6.00 ± 0.00	28.25 ± 0.82
*S. aureus* SA 02/10	6.00 ± 0.00	6.00 ± 0.00	12.00 ± 0.00	14.00 ± 0.00	16.00 ± 1.41	14.50 ± 0.71	13.00 ± 0.00	16.50 ± 0.71	16.00 ± 1.41	14.00 ± 1.41	17.00 ± 1.41	17.00 ± 1.41	6.00 ± 0.00	28.50 ± 0.60
*S. aureus* SA 03/10	6.00 ± 0.00	6.00 ± 0.00	6.00 ± 0.00	10.00 ± 1.41	14.50 ± 0.71	11.50 ± 0.71	9.50 ± 0.71	13.00 ± 1.41	10.50 ± 0.71	11.00 ± 1.41	13.50 ± 0.71	15.00 ± 0.00	6.00 ± 0.00	20.00 ± 0.50
*S. aureus* SA 08	6.00 ± 0.00	6.00 ± 0.00	8.50 ± 0.71	19.00 ± 0.00	20.00 ± 0.00	18.00 ± 0.00	17.00 ± 1.41	21.00 ± 1.41	19.50 ± 0.71	17.00 ± 0.00	20.50 ± 0.71	20.50 ± 0.71	6.00 ± 0.00	25.33 ± 0.58
MRSA 10/08	6.00 ± 0.00	6.00 ± 0.00	11.00 ± 1.41	16.50 ± 0.71	15.00 ± 1.41	14.00 ± 1.41	13.00 ± 1.41	15.50 ± 0.71	14.00 ± 1.41	13.00 ± 1.41	16.00 ± 0.00	16.00 ± 1.41	6.00 ± 0.00	15.20 ± 0.50
MRSA 12/08	6.00 ± 0.00	6.00 ± 0.00	11.50 ± 0.71	16.00 ± 1.41	10.00 ± 1.41	12.00 ± 0.00	15.50 ± 0.71	17.00 ± 1.41	14.00 ± 0.00	15.00 ± 1.41	20.00 ± 0.00	15.00 ± 1.41	6.00 ± 0.00	12.25 ± 0.60
**Gram-negative Bacteria**														
*E. coli* ATCC 25922	6.00 ± 0.00	6.00 ± 0.00	6.00 ± 0.00	6.00 ± 0.00	6.00 ± 0.00	6.00 ± 0.00	6.00 ± 0.00	6.00 ± 0.00	6.00 ± 0.00	6.00 ± 0.00	6.00 ± 0.00	6.00 ± 0.00	6.00 ± 0.00	23.25 ± 0.50
*P. aeruginosa* ATCC 27853	6.00 ± 0.00	6.00 ± 0.00	6.00 ± 0.00	6.00 ± 0.00	6.00 ± 0.00	6.00 ± 0.00	6.00 ± 0.00	6.00 ± 0.00	6.00 ± 0.00	6.00 ± 0.00	6.00 ± 0.00	6.00 ± 0.00	6.00 ± 0.00	11.50 ± 0.58
*K. pneumoniae* ATCC 13883	6.00 ± 0.00	6.00 ± 0.00	6.00 ± 0.00	13.00 ± 1.41	11.00 ± 1.41	9.00 ± 1.41	13.00 ± 1.41	16.00 ± 1.41	15.00 ± 1.41	14.00 ± 1.41	16.50 ± 0.71	16.00 ± 1.41	6.00 ± 0.00	22.25 ± 0.50
**Yeasts**													**DMSO**	**Amphotericin B**
*C. albicans* ATCC 90028	6.00 ± 0.00	6.00 ± 0.00	6.00 ± 0.00	14.50 ± 0.71	17.00 ± 0.00	11.00 ± 1.41	11.50 ± 0.71	16.00 ± 0.00	10.50 ± 0.71	13.00 ± 1.41	18.00 ± 0.00	11.50 ± 0.71	6.00 ± 0.00	20.33 ± 0.58
*C. tropicalis* ATCC 750	10.00 ± 0.00	7.00 ± 1.41	6.50 ± 0.71	21.00 ± 1.41	21.00 ± 1.41	16.50 ± 0.71	15.00 ± 0.00	20.00 ± 0.00	19.00 ± 1.41	20.50 ± 0.71	24.00 ± 0.00	20.00 ± 0.00	6.00 ± 0.00	21.50 ± 0.58

**Table 5:** MIC values (mg/mL) of *E. glubulus* extracts (Modal values).

Strains	Ethanol			Methanol			75% Ethanol			Controls	
	Wood	Stump Wood	Stump Bark	Wood	Stump Wood	Stump Bark	Wood	Stump Wood	Stump Bark	DMSO (%)	Tetracycline (µg/mL)
**Gram-positive Bacteria**											
*S. aureus* ATCC 25923	1.25	1.25	0.08	1.25	1.25	0.156	2.5	1.25	0.02	>20	0.06
*B. cereus* ATCC 11778	0.156	0.156	0.156	0.313	0.156	0.313	0.313	0.156	0.04	>20	0.06
*L. monocytogenes* LMG 16779	2.5	1.25	0.08	2.5	1.25	0.156	5	0.625	0.02	>20	0.06
*E. faecalis* ATCC 29212	>10	>10	2.5	>10	>10	2.5	>10	>10	0.313	>20	0.06
*S. aureus* SA 01/10	5	1.25	0.313	2.5	1.25	0.313	5	2.5	0.156	>20	0.12
*S. aureus* SA 02/10	1.25	2.5	0.156	2.5	0.625	1.25	2.5	2.5	0.156	>20	0.12
*S. aureus* SA 03/10	>10	>10	>10	>10	>10	>10	>10	>10	>10	>20	0.24
*S. aureus* SA 08	1.25	0.625	1.25	2.5	0.625	1.25	5	0.625	0.625	>20	0.12
MRSA 10/08	2.5	1.25	0.313	5	1.25	0.625	5	1.25	0.156	>20	0.48
MRSA 12/08	>10	>10	1.25	>10	>10	2.5	>10	>10	0.156	>20	0.48
**Gram-negative Bacteria**											
*E. coli* ATCC 25922	5	2.5	5	10	5	5	10	2.5	5	>20	0.06
*P. aeruginosa* ATCC 27853	10	5	10	10	5	10	10	5	5	>20	0.24
*K. pneumoniae* ATCC 13883	1.25	0.625	2.5	2.5	0.625	2.5	2.5	0.625	1.25	>20	0.06
**Yeasts**										**DMSO (%)**	**Amphotericin B (µg/mL)**
*C. albicans* ATCC 90028	0.08	0.02	0.04	0.08	0.02	0.04	0.08	0.02	0.04	>20	0.25
*C. tropicalis* ATCC 750	0.156	0.08	0.156	0.313	0.04	0.156	0.156	0.08	0.08	>20	0.50

Generally, the Gram-positive strains present lower MIC values than Gram-negative and yeast strains which indicates that Gram-positive bacteria were the most susceptible strains to the extracts. The difference in susceptibility between Gram-negative and Gram-positive bacteria to inhibition by plant extracts is supported by other researchers [37,38]. It is not known exactly why Gram-negative bacteria should be less susceptible but it may be related to the outer membrane of Gram-negative bacteria which endows the bacterial surface with strong hydrophilicity and acts as a strong permeability barrier [38]. The morphological differences between Gram-positive and Gram-negative bacteria could justify the different sensitivity, namely because Gram-negative bacteria have an outer phospholipidic membrane carrying the structural lipopolysaccharide components, which cannot be found in Gram-positive bacteria. This makes the cell wall impermeable to lipophilic solutes, while porins constitute a selective barrier to the hydrophilic solutes with an exclusion limit of about 600 Da [37]. Another explanation may be the inhibition of the peptidoglycan synthesis in Gram-positive bacteria, by the compounds present in the extracts [37]. It is possible to conclude that biomass from *E. globulus*, particularly the stumps, are rich in molecules with antimicrobial properties, which can be potentially used in new formulations to prevent microbial growth.

## 3. Experimental Section

### 3.1. Raw Material

The stump material (industrial chips) used in this study were randomly collected from a crushed stump pile from ALTRI (Vila Velha de Ródão, Portugal); these chips are used as raw-material for pulp and paper. The material was immediately processed after stabilization of moisture content in an oven (60 °C). Additional industrial wood chips (average dimension of 8 mm × 3 mm × 2 mm), and stumps bark of *Eucalyptus globulus*, kindly provided by the same company, were fractionated using a knife mill, reduced to coarse powder and screened to a size ranging between 2–10 mm.

### 3.2. Extraction Process

The coarse powder obtained from the three raw materials was extracted using a Soxhlet apparatus (*ca.* 1 L) with *n*-hexane, ethanol and methanol as solvents. Hydroalcoholic extractions (ethanol/water 75:25; v/v), were performed by refluxing, using 100 g of each raw material sample with 1000 mL of solvent. All extracts were concentrated in a rotary evaporator and further dried in a vacuum oven at 35 °C and 150 mbar. Each extraction was repeated two times and the extraction yields were determined gravimetrically by weighing the solids before and after the extraction. Then, 5 mL of each extract was diluted in 45 mL of methanol for phytochemical screening and antioxidant activity evaluation. For disc diffusion assay, the extracts were dissolved in pure dimethyl sulfoxide (DMSO) and for MIC determination the extracts were dissolved in culture medium with maximum 10% DMSO.

### 3.3. Total Phenolic Compounds Determination

The phenolics were determined by Folin-Ciocalteu’s colorimetric method. The methanolic solutions of each extract (50 μL) or gallic acid (standard phenolic compound) were mixed with distilled water (450 μL), and then 0.2 N Folin-Ciocalteu’s reagent (2.5 mL, diluted with distilled water) was added. The mixtures were allowed to stand for 5 min, and then aqueous Na_2_CO_3_ (2 mL, 75 g/L) was added. After incubation of these reaction mixtures (90 min/30 °C), the total phenols were determined by colorimetry at 765 nm. The standard curve was prepared using 500, 400, 350, 325, 300, 250, 225, 200, 150, 125, 100, and 50 mg/L solutions of gallic acid in methanol (y = 0.0010 x; R^2^ = 0.9800). Total phenolic values were expressed as gallic acid equivalents (mg GAE/g of extract), which is a common reference compound for phenolic compounds [28]. The tests were conducted in triplicate.

### 3.4. Tannin Content

Tannins were measured as the difference in total phenolics (measured by Folin-Ciocalteu’s reagent) before and after treatment with insoluble polyvinylpolypyrrolidone (PVPP), as this polymer binds strongly to tannins [39]. Each methanolic extract (1 mL) was added to an aqueous solution of PVPP (1 mL, 70 mg/mL). After vigorous shaking, the samples remained for 15 min at 4 °C, to develop the tannin-PVPP complex [25,40]. Then, the samples were centrifuged for 10 min at 3000 rpm; the tannins were found in the residue and the free phenols/non-adsorbed phenolics in the supernatant. Total phenols in the supernatant were determined by the Folin-Ciocalteu’s colorimetric method, as described above, and the concentration of tannins was calculated as the difference between the total phenols and free phenols, and is expressed in their respective units (mg GAE/g of extract) [25,40]. These determinations were made in triplicate.

### 3.5. Flavonoids Determination

The aluminum chloride colorimetric method was used to determine the flavonoids content according to a previously implemented method [23,28]. Each methanolic solution of the extracts (500 μL) was separately mixed with methanol (1.5 mL), 10% aluminum chloride (0.1 mL), 1 M potassium acetate (0.1 mL), and distilled water (2.8 mL). This solution remained at room temperature for 30 min; the absorbance of the reaction mixture was measured at 415 nm using a spectrophotometer. The calibration curve was constructed by preparing eight quercetin solutions at concentrations ranging from 12.5 to 200 μg/mL in methanol (y = 0.0078 x; R^2^ = 0.9994). Total flavonoid values were expressed as quercetin equivalents (mg QE/g of extract) [23,28]. These determinations were made in duplicate.

### 3.6. Evaluation of Antioxidant Activity

#### 3.6.1. DPPH Scavenging Assay

The antioxidant activity of the extracts and standards (gallic acid and quercetin) was determined by the radical scavenging activity method using the 2,2-diphenyl-1-picrylhydrazyl (DPPH) radical [32]. Briefly, aliquots of methanolic solutions of the extracts or standards (0.1 mL) at different concentrations were added to a DPPH methanolic solution (3.9 mL). Three DPPH solutions were tested, 0.2000, 0.1242 and 0.0800 mM, which were prepared by dissolving 39.4, 24.5 and 15.8 mg in 500 mL of methanol, respectively. These concentrations were selected due to the linearity range of DPPH solutions: above 0.2 mM the concentration is very high, and below 0.5 mM the color is very weak having a limited range of absorbance reading. The control sample consisted of a solution of 0.1 mL of methanol mixed with 3.9 mL of DPPH. After a 90 min incubation period at room temperature in the dark, the absorbance was measured at 517 nm.

The radical scavenging activity was calculated as follows: I% = [(Abs_0_ − Abs_1_)/Abs_0_] × 100, where Abs_0_ was the absorbance of the control and Abs_1_ was the absorbance in the presence of the test sample at different concentrations. The IC_50_ was calculated graphically using a calibration curve in the linear range, by plotting the extract concentration *vs.* the corresponding scavenging effect. The antioxidant activity was expressed as the Antioxidant Activity Index (AAI), calculated as follows: AAI = (final concentration of DPPH in the control sample)/(IC_50_) [32]. Thus, the AAI was calculated considering the mass of DPPH and the mass of the tested sample in the reaction, resulting in a constant for each sample, independent of the concentration of DPPH and sample used. In this work, it was considered that the extracts showed poor antioxidant activity when AAI < 0.5, moderate antioxidant activity between 0.5 and 1.0, strong antioxidant activity between 1.0 and 2.0, and very strong when AAI > 2.0 [32]. Assays were carried out in duplicate and all DPPH solutions were prepared daily.

#### 3.6.2. β-Carotene Bleaching Test

After preparation of β-carotene solution (20 mg/mL in chloroform), 20 μL was added to linoleic acid (40 μL), Tween 40 (400 mg) and chloroform (1 mL). This mixture was then evaporated at 45 °C for 5 min in a rotary vacuum evaporator to remove chloroform and immediately diluted with oxygenated distilled water (100 mL). The water was added slowly to the mixture and vigorously agitated to form an emulsion. Then, this emulsion (5 mL) was transferred to test tubes containing the extracts in methanol at different concentrations (300 μL). About 5 mL of the emulsion and 300 μL of samples in methanol were used as control. Standard butylated hydroxytoluene (BHT) in methanol, at the same concentration as samples, was used as reference. The tubes were then gently shaken and placed at 50 °C in a water bath for 2 h. The absorbances of the extracts, standard and control were measured at 470 nm, using a spectrophotometer, against a blank consisting of an emulsion without β-carotene. The measurements were carried out at initial time (t = 0 h) and at final time (t = 2 h). The antioxidant activity was measured in terms of percentage of inhibition of β-carotene’s oxidation by: % Inhibition = (Abs^t=2^_sample_ − Abs^t=2^_control_)/(Abs^t=0^_control_ − Abs^t=2^_control_). Where Abs^t=2^ was the absorbance of the sample or control at final time of incubation and Abs^t=0^ was the absorbance in the control at initial time of incubation [24].

### 3.7. Determination of Antimicrobial Activity

#### 3.7.1. Test Microorganisms and Culture Media

The antimicrobial studies were carried out against thirteen bacterial strains (Gram-positive: *Staphylococcus aureus* ATCC 25923, *Bacillus cereus* ATCC 11778, *Listeria monocytogenes* LMG 16779 and *Enterococcus faecalis* ATCC 29212; Gram-negative: *Escherichia coli* ATCC 25922, *Pseudomonas aeruginosa* ATCC 27853, and *Klebsiella pneumoniae* ATCC 13883; Clinical isolates of *S. aureus*: SA 01/10, SA 02/10, SA 03/10 and SA 08; Clinical methicillin-resistant *S. aureus*: MRSA 10/08 and MRSA 12/08) and two yeast strains (*Candida albicans* ATCC 90028 and *Candida tropicalis* ATCC 750). Stock cultures were prepared and stored with 20% glycerol at −80 °C. The strains were sub-cultured on an appropriate agar plate 24 h prior to any antimicrobial test and when cultured from stock, they were sub-cultured before use. Brain Heart Infusion Agar (BHI) was used for the growth of bacterial species and Sabouraud Dextrose Agar (SDA) was used for yeasts.

#### 3.7.2. Disc Diffusion Assay

Antimicrobial activity of the extracts was determined by disc diffusion assay, using either the M2-A8 method, described by Clinical Laboratory and Standards Institute (CLSI), for bacteria or the M44-A2 method described by CLSI for yeasts. For the preparation of inoculum, bacteria or fungi were suspended in saline solution to a cell suspension of 0.5 McFarland (about 1 to 2 × 10^8^ colony-forming unit/mL (CFU/mL) to non-fastidious bacteria and 1 to 5 × 10^6^ CFU/mL for yeasts). The discs (6 mm diameter) were impregnated with 20 μL of each extract (4 mg/disc) at a concentration of 200 mg/mL and placed on the inoculated agar. Negative controls were prepared using DMSO as it had been previously used to dissolve the extracts. Positive controls were prepared using tetracycline (30 µg/disc) in the case of bacteria and amphotericin B (25 µg/disc) in the case of yeasts. The plates inoculated with non-fastidious bacteria were incubated at 37 °C for 24 h and for 48 h in the case of yeasts. After incubation, all plates were checked for inhibition zones and the diameters were measured in millimeters. All experiments were carried out in triplicate [41].

#### 3.7.3. Resazurin Microtiter Method

The antimicrobial activity of each extract was assessed using resazurin microtiter assay. Plates were prepared under aseptic conditions. A sterile 96 well plate was labeled. In the case of bacteria, a volume of 100 µL of extract in 10%, v/v, DMSO-stock concentration of 20 mg/mL in Müeller-Hinton Broth (MHB), the maximum DMSO concentration tested was 10%-was pipetted into the first row of the plate. To all other wells 50 µL of MHB was added. Serial dilutions were performed using a multichannel pipette. Tips were discarded after use such that each well had 50 µL of the test material in serially descending concentrations. To each well, resazurin indicator solution (10 µL, 0.1% diluted in MHB) was added. Using a pipette, fresh MHB (30 µL) was added to each well. Finally, bacterial suspension (10 µL, 0.5 McFarland) was added to the wells. Each plate was wrapped loosely with cling film to ensure that bacteria did not become dehydrated, and had a set of controls: a column with a broad-spectrum antibiotic as a positive control, a column with all solutions with the exception of the test compounds, and a column with all solutions with the exception of the bacterial suspension, adding the respective volume of MHB instead. The plates were prepared in triplicate, and placed in an incubator set at 37 °C for 18 h [42,43]. For yeasts, inoculum was prepared by transferring several colonies to sterile saline solution in order to achieve turbidity of a 0.5 McFarland. Resazurin was also employed as an indicator of cell growth. For this purpose, the working suspension (inoculum diluted 1:1000 in culture media) was supplemented with resazurin sterilized solution (50 µL, 20 mg/mL in water). Broth medium used for this test was prepared as follows: RPMI-1640 medium (5.215 g), supplemented with glutamine and phenol red, without bicarbonate and 3-(*N*-morpholino)propanesulfonic acid (MOPS, 35.53 g) were dissolved in distilled water (400 mL), adjusting the pH to 6.9–7.1 at 25 °C with 1 mol/L sodium hydroxide. Additional water was added to bring the medium to a final volume of 0.5 L, which was filter sterilized and stored at 4 °C until required. Microdilution susceptibility testing was performed, as it was described above for bacteria, changing the final volume in the wells of the microplate to 200 µL. The plates were prepared in triplicate, and placed in an incubator set at 37 °C for 24 h [44]. The color change was then assessed visually. Any color changes from purple to pink or colorless were recorded as positive. The lowest concentration at which change occurred was taken as the Minimum Inhibitory Concentration (MIC) value [42,43,44].

### 3.8. Statistical Analysis

Results are presented as mean values ± standard deviation or as modal modal values. In order to determine the measurements reproducibility, each assay was done in duplicate or triplicate. Relative Standard Deviation of all measurements was lower than 10%. *p* < 0.01 was assumed as statistical difference between experimental points.

## 4. Conclusions

The results obtained in this work showed the presence of high concentrations of total phenolic compounds and flavonoids in extracts of *Eucalyptus globulus* stumps. A positive linear correlation between Antioxidant Activity Index and total phenolic content was observed for all the extracts studied (R^2^ = 0.9636). Generally the stump wood extracts stand out from the other ones, presenting the highest percentages of inhibition of linoleic acid oxidation, closely similar to those of the synthetic antioxidant BHT. It was also possible to conclude that Gram-positive bacteria, including clinical isolates of *S. aureus* and MRSA strains, were more susceptible to the action of the extracts.

The investigations into the bioactive properties of *E. globulus* stumps have revealed that they can be used as an important source for the extraction of molecules with antioxidant and antimicrobial activities, particularly polyphenolic compounds, which are well recognized potent bioactive compounds. Further work is in progress to purify the extract that gave the best biological activities in order to identify the molecule(s) responsible for those properties.
